# A GSH/CB Dual-Controlled Self-Assembled Nanomedicine for High-Efficacy Doxorubicin-Resistant Breast Cancer Therapy

**DOI:** 10.3389/fphar.2021.811724

**Published:** 2022-01-14

**Authors:** Yang Yang, Quanfeng Zhao, Zhe Peng, Yunjiang Zhou, Miao-Miao Niu, Lin Chen

**Affiliations:** ^1^ Department of Pharmacology, Chongqing Health Center for Women and Children, Chongqing, China; ^2^ Department of Pharmacy, Southwest Hospital, First Affiliated Hospital to TMMU, Third Military Medical University (Army Medical University), Chongqing, China; ^3^ Key Laboratory of Drug Quality Control and Pharmacovigilance, Ministry of Education, State Key Laboratory of Natural Medicines, School of Basic Medicine and Clinical Pharmacy, China Pharmaceutical University, Nanjing, China; ^4^ Department of Pharmacology, Chongqing Medical University, Chongqing, China

**Keywords:** cyclic peptide, GSH, cathepsin B, drug-resistance, breast cancer

## Abstract

Chemoresistance is a major therapeutic obstacle in the treatment of breast cancer. Therefore, how to overcome chemoresistance is a problem to be solved. Here, a glutathione (GSH)/cathepsin B (CB) dual-controlled nanomedicine formed by cyclic disulfide-bridged peptide (cyclic-1a) as a potent anticancer agent is reported. Under the sequential treatment of GSH and CB, cyclic-1a can efficiently self-assemble into nanofibers. *In vitro* studies show that cyclic-1a promotes the apoptosis of MCF-7/DOX cells by inducing the cleavages of caspase-3 and PARP. *In vivo* studies confirm that cyclic-1a significantly inhibits the progression of MCF-7/DOX cells-derived xenograft in nude mice, with no obvious adverse reactions. This study provides a paradigm of GSH/CB dual-controlled nanomedicine for high-efficacy and low-toxic DOX-resistant breast cancer therapy.

## Introduction

The cancer burden caused by breast cancer is increasing rapidly. Despite some progression in treatment, breast cancer remains the most commonly diagnosed cancer and the leading cause of cancer death for women worldwide, with 2.3 million estimated new cases and almost 685,000 deaths in 2020 (Sung et al., 2021). Doxorubicin (DOX), a broad-spectrum antibiotic and anticancer drug, has been used to treat a variety of hematological and solid malignancies including breast cancer, owing to its superior therapeutic efficacy (Liang et al., 2019; Wallace et al., 2020). In breast cancer therapy, DOX is one of the most frequently used chemotherapeutic agents. However, its clinical application is largely limited by acquired resistance and serious adverse reactions (Li et al., 2019; Liang et al., 2019). Therefore, there is an urgent need to develop a novel class of safe and effective therapeutic agents for overcoming drug-resistance in breast cancer.

Therapeutic resistance leading to tumor relapse is a major obstacle to breast cancer chemotherapy (BeLow and Osipo, 2020; Wu et al., 2020). It is well known that overexpression of glutathione (GSH) is closely related to the development of chemoresistance in a variety of human malignancies, including breast cancer, colon cancer, and lung cancer (Jiang et al., 2017; Kennedy et al., 2020). GSH may promote the development of drug-resistance by conjugating chemotherapeutic agents to facilitate their efflux, or by counteracting the prooxidant effects of anticancer drugs, favoring the repair processes of DNA damages (Jiang et al., 2017; Bansal and Simon, 2018). Prior reports have demonstrated that GSH is overexpressed in drug-resistant cells of breast cancer and other cancers (Bansal and Simon, 2018). Cathepsin B (CB) is a lysosomal acid cysteine protease which is of great significance in tumor progression and drug-resistance (Withana et al., 2012; Shim et al., 2016). Recent studies suggest that overexpression of CB is closely associated with chemoresistance and poor prognosis in breast cancer cells (Vasiljeva et al., 2008; Bengsch et al., 2014; Rizzo et al., 2020; Shim et al., 2020; Wang et al., 2020). Using the reactions of GSH or CB to control self-assembly have attracted extensive attention. For example, the groups of Ling and Farokhzad have described GSH-responsive self-assembled nanoparticles for effective drug delivery and cancer therapy (Ling et al., 2019). Zhang and co-workers recently described the control of a molecular self-assembly by CB, which showed potent cytotoxicity against tumor cell lines (Zhang et al., 2016). Shim and co-workers also developed nanoparticles of CB-cleavable peptide-conjugated prodrug that induced cytotoxicity against CB-overexpressed tumor cells (Shim et al., 2019). However, research efforts mainly focus on using a single-step reaction catalyzed by a CB or GSH to trigger the self-assembly process. Although single-controlled self-assembly have showed promising applications in cancer therapy and drug delivery, multistep self-assembly can lead to more sophisticated and functional materials (Hnilova et al., 2012; Lin et al., 2019; Wee et al., 2019; Chen et al., 2020; Song et al., 2020). Previous literatures reported multifunctional nanoparticles for both drug-sensitive and drug-resistant breast cancer therapy (Kang et al., 2010; Li et al., 2010; Shieh et al., 2011). Compared with these previous developed nanostructures, nanostructures formed by peptides have attracted research interest, because peptides have several unique advantages such as modular design and easiness of synthesis, low immunogenicity and toxicity, good biocompatibility, convenient modification with known functional motifs, and rapid responses to various external stimuli (Terech et al., 2006; Yan et al., 2010; Leite et al., 2015; Arnon et al., 2016; Cai et al., 2016; Mathur et al., 2021). Due to the higher levels of both GSH and CB in drug-resistance cancer cells, we speculated that peptide nanostructures formed by GSH/CB dual-controlled self-assembly might have high selectivity and therapeutic efficacy towards drug-resistant cancer cells, low toxicity and good biocompatibility.

Enzyme-triggered self-assembly and hydrogelation of linear peptides have attracted a great deal of attention due to their good biocompatibility (Qin et al., 2013; Vigier-Carrière et al., 2015; Abbas et al., 2017; Wang et al., 2017). Upon undergoing self-assembly at sufficient concentration, peptide-based nanofibers form an entangled network which reduces the flow of solvents, leading to the hydrogel state (Nguyen et al., 2018; King et al., 2016; O'Leary et al., 2011; Chen et al., 2019). Such enzyme-triggered peptide self-assembly provides an efficient and practical method for controlling nanofiber self-assembly and hydrogelation as a function of environment (Rudra et al., 2010). Linear peptides have been designed to undergo self-assembly in response to enzymatic catalysis in biological microenvironments (Rudra et al., 2010; Rudra et al., 2012). As a useful strategy for generating supramolecular nanofibers/hydrogels, linear peptide-based hydrogelations show promising applications in cell fate control, drug delivery, immune modulation, biosensing, and regenerative medicine (Habibi et al., 2016; Acar et al., 2017; Zhou et al., 2021). Despite many advantages of linear peptides as the precursors for enzyme-triggered hydrogelation, linear peptides are easily degraded by extracellular serum proteases before entering the cytoplasm of cells (Lian et al., 2014; Yang et al., 2020), which greatly restricts their clinical application.

Recently, self-assembly of cyclic disulfide-bridged peptides are emerging as a powerful method for controlling self-assembly as a function of tumor microenvironment (Yang et al., 2020). Among the strategies for enhancing peptide biostability, the introduction of a conformational constraint by peptide disulfide-bridged cyclization is an effective one (Yang et al., 2007; Carlini et al., 2019; Yang et al., 2020). This unique feature of conformational constraints endows cyclic disulfide-bridged peptides with long-term biostability because of their resistance against various proteases *in vitro* and *in vivo*. The previous study has proved that disulfide-bridged cyclization of the redox-triggered peptide confers both improved proteolytic biostability and enhanced intracellular delivery efficiency, while the reversibility of the disulfide bond permits the release of functional linear peptides in the reducing microenvironment of the tumor cells (Yang et al., 2020). Compared with redox-triggered self-assembly of peptides, GSH/CB dual-controlled self-assembly of cyclic disulfide-bridged peptides might lead to more sophisticated and functional biomaterials. Therefore, we envisaged that using intracellular GSH and CB reactions to trigger molecular self-assembly of cyclic disulfide-bridged peptides could generate superior active biomedical nanomaterials.

**FIGURE 1:**
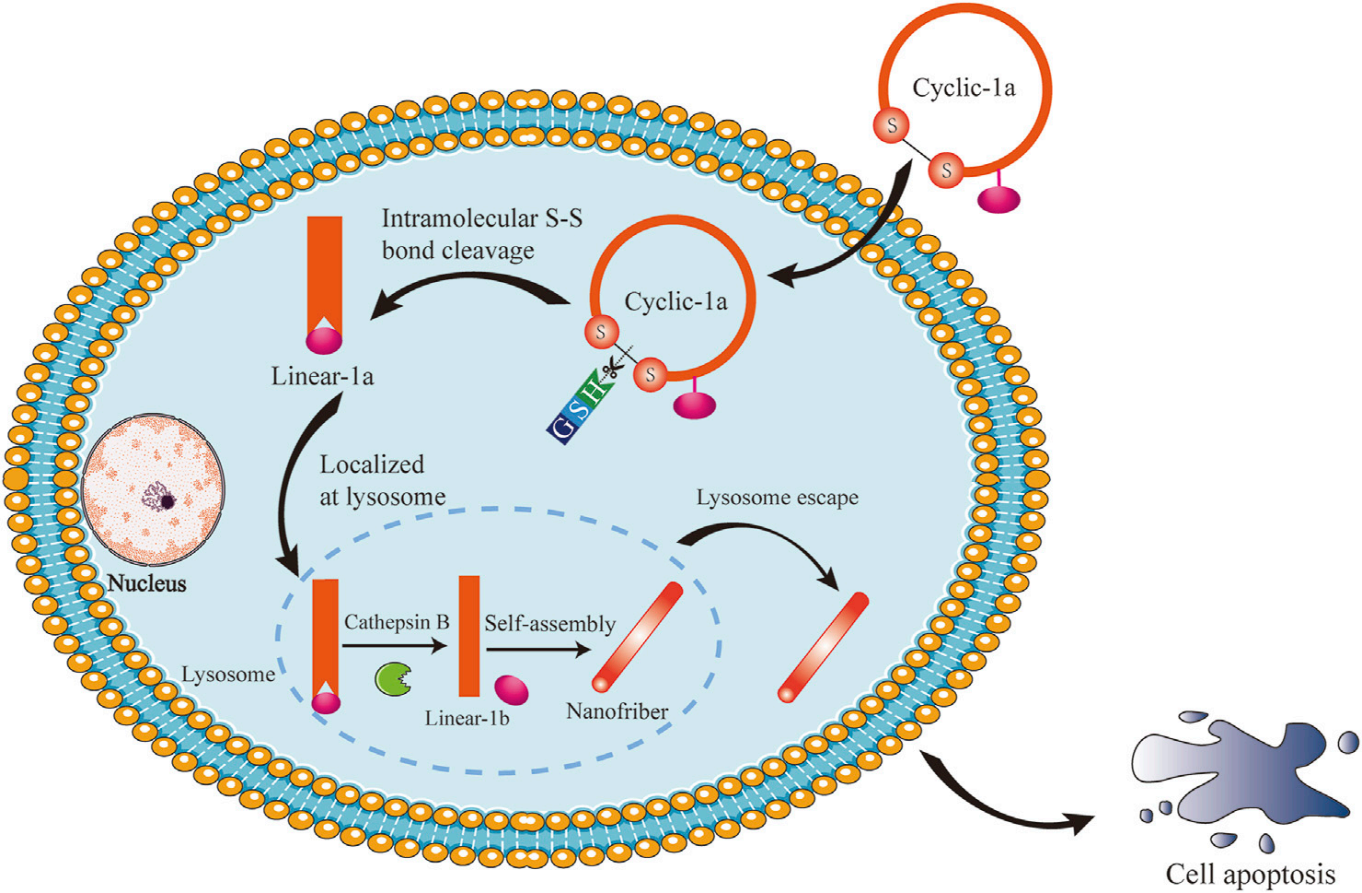
Schematic illustration of intracellular GSH/CB dual-controlled self-assembly behavior of the cyclic peptide precursor that lead to apoptosis of DOX-resistant breast cancer cells.

Here, we reported an intracellular GSH/CB dual-controlled supramolecular nanomedicine, cyclic-1a. Upon entering the cytosol of MCF-7/DOX cells by a diffusion process, the precursor cyclic-1a self-assembled into nanofibers under the sequential treatment of GSH and CB (Figure 1). when undergoing self-assembly at sufficient concentration, supramolecular nanofibers induced intracellular hydrogelation, leading to cell apoptosis through the PARP/caspase-3 signaling pathway. Therefore, cyclic-1a might be used as a potential chemotherapeutic agent for DOX-resistant breast cancer.

## Materials and Methods

See Supplementary Material for more details.

## Results and Discussion

### Synthesis and Characterization of the Supramolecular Gelator Precursor (Cyclic-1a)

Intracellular stimuli-responsive nanofibers generally should have several features in common (Yang et al., 2020): (i) building blocks self-assemble to exclusively form nanofibers inside cells; (ii) a self-assembly process of intracellular nanofibers is triggered by intracellular stimuli; and (iii) the formation of nanofibers induces observable cellular phenomena. Thus, to demonstrate the general concept, we opt to use a GSH/CB dual-controlled formation of nanofibers and supramolecular hydrogelation inside DOX-resistant breast cancer cells. As shown in Figure 1, a cyclic peptide gelator precursor (termed as cyclic-1a) was constructed via disulfide bonding of the flanking cysteine residues. Upon entering the cytosol of cells, the disulfide bond of cyclic-1a was reduced by intracellular GSH to produce its linear conformation linear-1a and then the lysosomal enzyme CB converted linear-1a into a peptide hydrogelator linear-1b that self-assembled into nanofibers. Eventually, the nanofibers would form intracellular supramolecular hydrogel and led to cell apoptosis.

To satisfy the design criteria illustrated in Figure 1, we designed the supramolecular gelator precursor cyclic-1a, the C_10_H_7_CH_2_C(O)CFFYKCGGRRGKGGHHHRRGDS sequence (disulfide bond cyclization). The cyclic-1a contains the following four distinct segments: 1) the naphthyl group (C_10_H_7_CH_2_-) for providing the hydrophobic force to improve the ability of self-assembly in aqueous environment (Zhou et al., 2021); 2) the tetrapeptide (FFYK) segment (being made of d-amino acids) being the major building block to act as both a donor and an acceptor of hydrogen bonds (Zhou et al., 2021); 3) the disulfided cysteine (Cys) motif as a trigger of GSH reduction, which was responsible for the first order nanofiber self-assembly; and 4) the peptide sequence (GGRRGKGGHHHRRGDS), whose cleavage by CB confers on the molecule the ability of the second order self-assembly.

**FIGURE 2:**
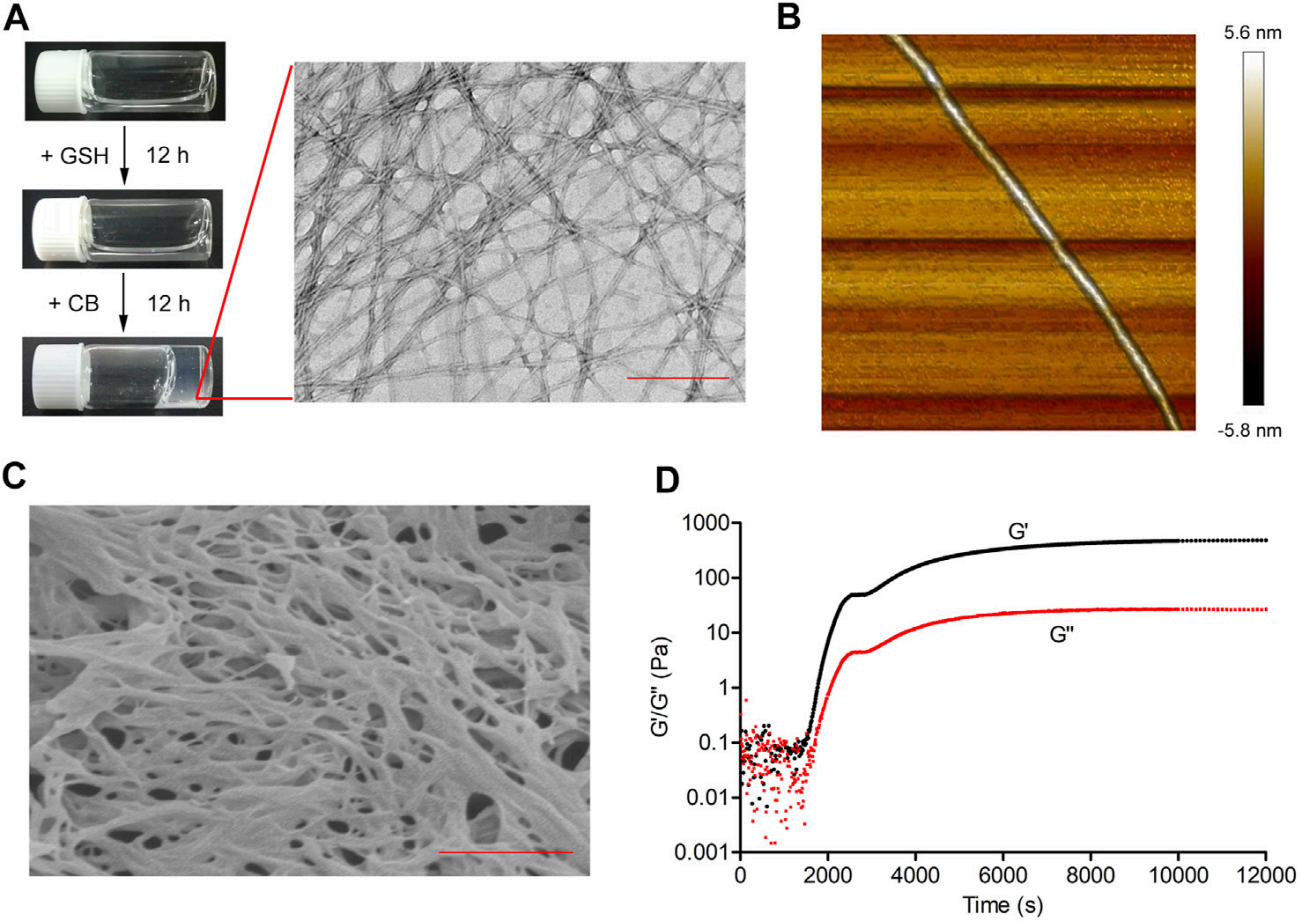
The self-assembly property of cyclic-1a. **(A)** Schematic illustration of the conversion of cyclic-1a into linear-1a by GSH and then of linear-1a into linear-1b by CB. Optical images of a solution of cyclic-1a (1,600 μM), a solution of cyclic-1a formed by adding GSH (8 mM) to a solution of linear-1a, and a gel of linear-1a formed by adding CB (1 U/mL) to a solution of linear-1b. TEM images of linear-1b (Scale bar = 200 nm). **(B)** AFM 3D-image of a single nanofiber. **(C)** SEM image of critical point dried hydrogel (Scale bar = 500 nm). **(D)** Oscillatory rheology of the cyclic-1a solution after the addition of both GSH and CB.

Next, we investigated the self-assembly properties of cyclic-1a. After addition of the GSH (8 mM) for 12 h, cyclic-1a solutions (1,600 μM) remained clear in phosphate-buffered saline (PBS; pH 7.4) (Figure 2A). The LC-MS analysis showed that more than 90% of cyclic-1a was converted into linear-1a at 7 h (Supplementary Figure S1). After 12 h, CB enzyme (1 U/mL) was added into the solution and the supramolecular hydrogel was formed for another 12 h (Figure 2A). The LC-MS analysis showed that more than 90% of linear-1a was converted into linear-1b at 20 h (Supplementary Figure S1). We next observed the characterization of linear-1b gels by the transmission electron micrograph (TEM), the atomic force microscopy (AFM) and the scanning electron microscopy (SEM). TEM was used to view the morphology and measure the length and width of structures on the nanoscale. TEM image of linear-1b gels showed that fiber diameters of fibrous networks were ∼7 nm (Figure 2A). To obtain direct topographical information, the gels were deposited on a mica surface and observed by AFM. The AFM 3D-images revealed that the height of the fiber was ∼4 nm (Figure 2B). The SEM image of linear-1b gels (Figure 2C) showed that a dense fiber network was apparent, directly complementing the fiber morphologies observed by TEM and AFM and also indicating the three-dimensional structure of the hydrogel. These results of TEM, AFM and SEM tests showed that after the addition of GSH and CB, the resulting linear-1b self-assembled into the nanofibers. Rheological tests (Figure 2D) showed that the hydrogelation started at 25 min after the GSH and CB were added at room temperature, as indicated by the storage modulus (G′) dominating the loss modulus (G″). This dual-responsive hydrogelation completed in 180 min, as indicated by the storage modulus (G′) reaching a plateau.

To test the biostability of cyclic-1a, the stability experiment was performed. Incubation of cyclic-1a in human serum for 360 min at 37°C resulted in ∼35% degradation, while the corresponding linear peptide, namely linear-L-1a, was completely degraded within 180 min under the same conditions (Supplementary Figure S2). These results suggest that cyclic-1a is more resistant to proteolytic hydrolysis than the corresponding linear peptide, making it a promising gelator precursor.

### *In Vitro* Anticancer Efficacy Against MCF-7/DOX Cells

**FIGURE 3:**
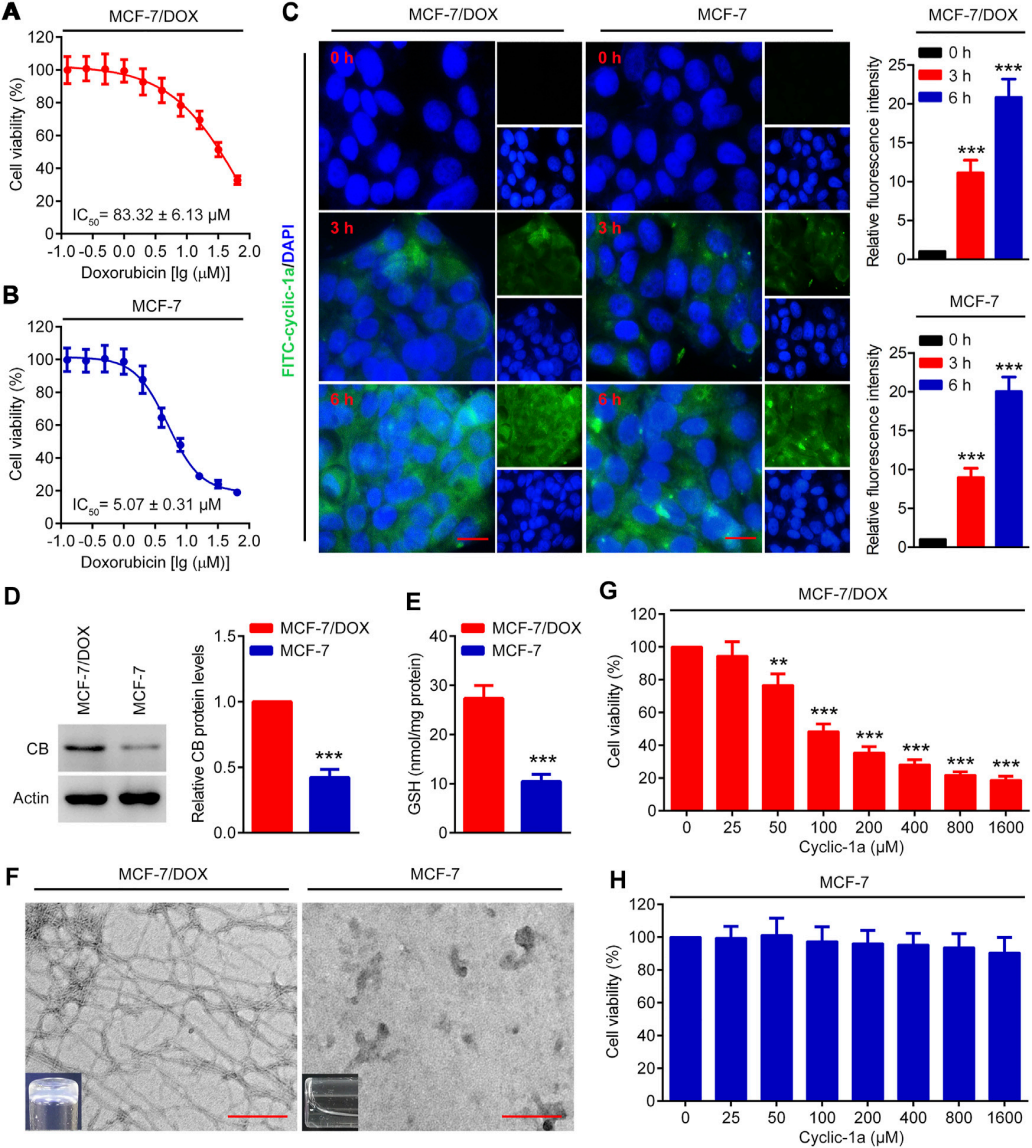
The cytotoxicity effects of cyclic-1a on MCF-7/DOX and MCF-7 cells. **(A–B)** The cytotoxicity effects of DOX on MCF-7/DOX and MCF-7 cells were detected by using MTT assay. **(C)** The cellular uptake ability of cyclic-1a. The images of MCF-7 and MCF-7/DOX cells treated with FITC-cyclic-1a. Scale bar = 10 μm. **(D)** The levels of CB in MCF-7/DOX and MCF-7 cells were detected by using western blot analysis. ***p < 0.001 vs MCF-7/DOX cells. **(E)** Intracellular GSH levels in MCF-7/DOX and MCF-7 cells. ***p < 0.001 vs MCF-7/DOX cells. **(F)** TEM observation and gelation test of the lysates of MCF-7/DOX and MCF-7 cells treated with 1,600 μM of cyclic-1a for 72 h (Scale bar = 200 nm). **(G–H)** The cytotoxicity effects of cyclic-1a on MCF-7/DOX and MCF-7 cells were detected by using MTT assay. **p < 0.01, ***p < 0.001 vs 0 μM of cyclic-1a group. Data are represented as mean ± SD, n = 3.

To investigate that MCF-7/DOX cells were doxorubicin-resistant, MTT assay was performed. As shown in Figures 3A,B, IC_50_ of MCF-7 cells to doxorubicin was 5.07 ± 0.31 μM, while IC_50_ of MCF-7/DOX cells to doxorubicin was 83.32 ± 6.13 μM, indicating that MCF-7/DOX cells were doxorubicin-resistant. Figure 3C showed that cyclic-1a had strong cellular uptake ability to MCF-7/DOX and MCF-7 cells. Subsequently, we detected the levels of CB and GSH in MCF-7/DOX and MCF-7 cells and found that the levels of CB and GSH in MCF-7/DOX cells were significantly higher than that in MCF-7 cells (Figures 3D,E). Next, we investigated the intracellular hydrogelation of cyclic-1a in these 2 cell lines and found that cyclic-1a could form the hydrogel in MCF-7/DOX cells, but not in MCF-7 cells (Figure 3F). TEM observation of the hydrogel suggested that the nanofibers with the width of ∼7 nm were morphologically similar to the nanofibers formed by cyclic-1a in aqueous solution (Figure 3F). These data suggest that the self-assembly of cyclic-1a is associated with high levels of both GSH and CB in MCF-7/DOX cells.

To further investigate the antiproliferative activity of cyclic-1a to MCF-7/DOX and MCF-7 cells, MTT assay was performed. As shown in Figure 3G, cyclic-1a inhibited the growth of MCF-7/DOX cells in a dose-dependent manner. However, cyclic-1a showed no obvious cytotoxicity to MCF-7 cells (Figure 3H). These results indicate that cyclic-1a can selectively inhibit the proliferation of MCF-7/DOX cells.

### Cyclic-1a Induces Cell Apoptosis of MCF-7/DOX Cells

**FIGURE 4:**
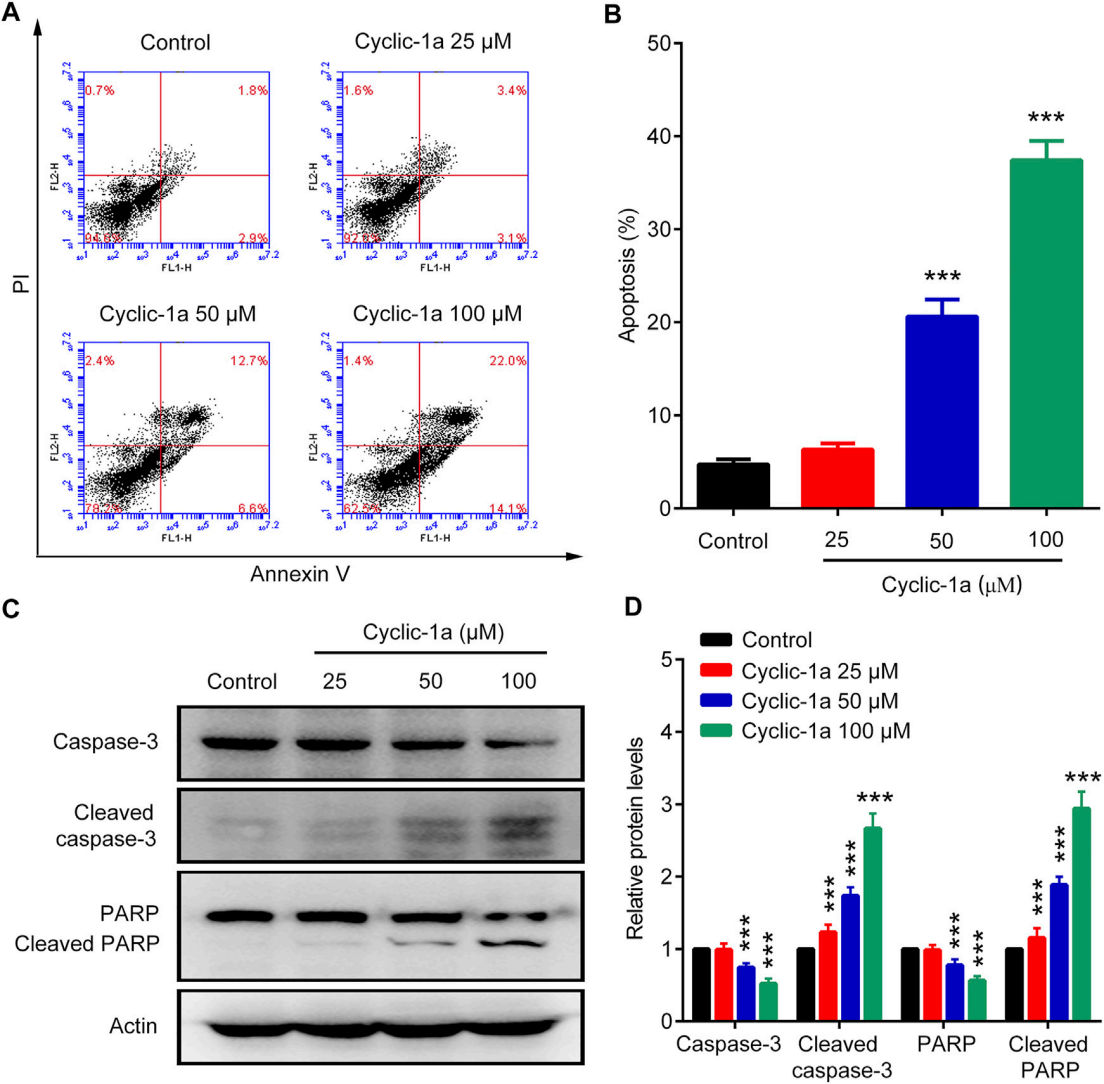
Effect of cyclic-1a on apoptosis of MCF-7/DOX cells. **(A–B)** Flow cytometry analysis. **(C–D)** Western blot analysis. Data are represented as mean ± SD, n = 3. ***p < 0.001 vs control group.

We also investigate the apoptosis rates of MCF-7/DOX cells treated with cyclic-1a. Figures 4A,B showed that various concentrations of cyclic-1a (25, 50, and 100 μM) promoted the apoptosis of MCF-7/DOX cells in a dose-dependent manner. Moreover, caspase-3 and PARP protein levels significantly down-regulated, while cleaved caspase-3 and cleaved PARP protein levels markedly up-regulated after treatment with cyclic-1a (Figures 4C,D). These results suggest that the formation of supramolecular hydrogel by the precursor cyclic-1a can efficiently induce the cleavages of caspase-3 and PARP, and then lead to cell apoptosis.

### *In Vivo* Anticancer Effect Against MCF-7/DOX Xenograft Tumors

**FIGURE 5:**
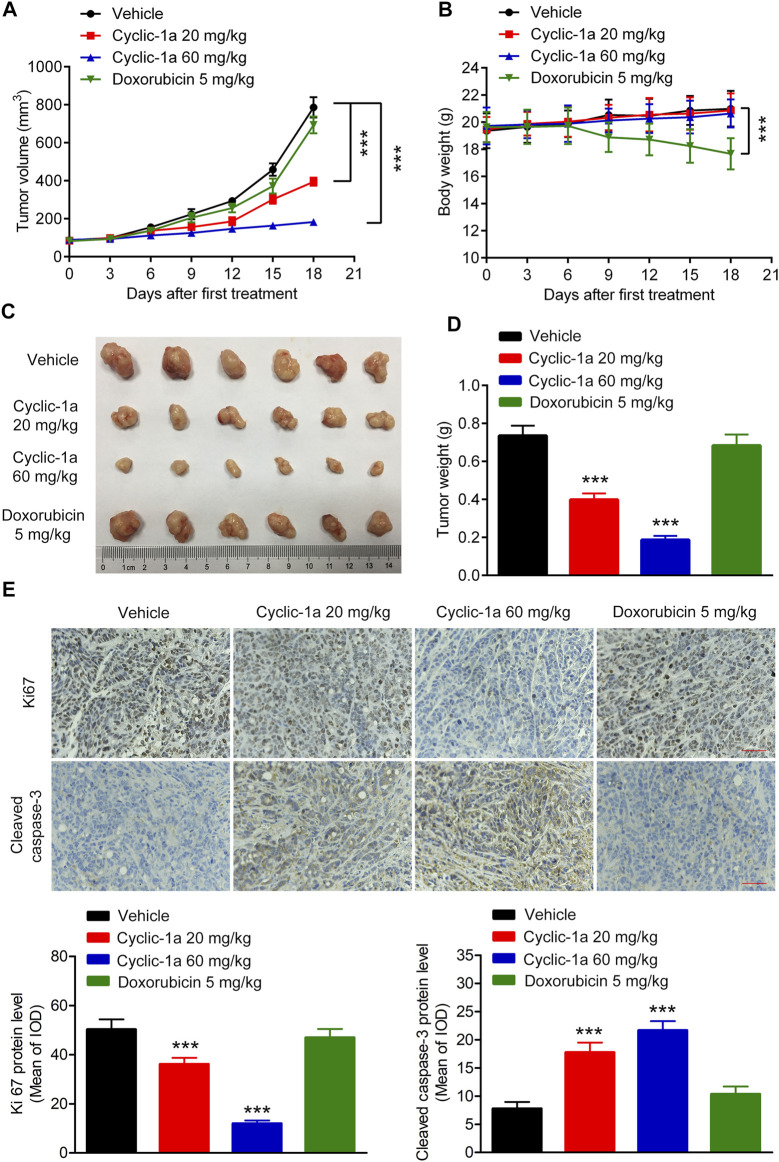
*In vivo* antitumor effect of cyclic-1a. **(A)** The tumor volume changes. **(B)** The body weight changes. **(C)** The image of tumors. **(D)** The tumor weights. **(E)** The protein levels of Ki67 and cleaved caspase-3 in tumors (Scale bar = 50 μm). Data are represented as mean ± SD, n = 6. ***p < 0.001 vs vehicle group.

**FIGURE 6:**
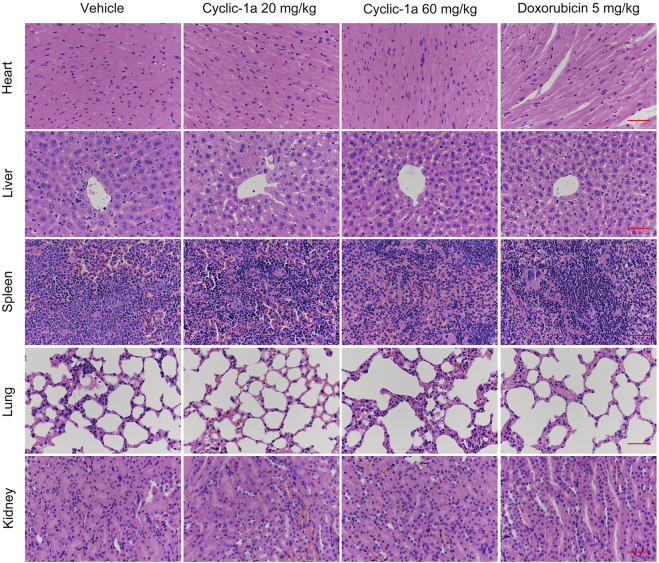
Morphological changes of organs in tumor-bearing mice (Scale bar = 50 μm).

Next, we tested the *in vivo* antitumor activity of cyclic-1a against MCF-7/DOX cells-derived xenograft. The model mice were intraperitoneally administrated with vehicle, cyclic-1a (20 mg/kg), cyclic-1a (60 mg/kg) and DOX (5 mg/kg). As shown in Figures 5A,C,D, cyclic-1a could slow the growth of the xenograft tumors. Moreover, immunohistochemistry analysis (Figure 5E) showed that the Ki67 levels were declined and cleaved caspase-3 levels were elevated after administrated with cyclic-1a. Moreover, body weight of mice gradually increased throughout the experiments except for the DOX-treated group (Figure 5B). Moreover, no significant pathological lesions were observed in the organs of the mice that administrated with cyclic-1a, whereas lesions of the heart and kidney were detected in the DOX treatment group (Figure 6). These results indicate that cyclic-1a displays high anticancer efficacy against MCF-7/DOX cells-derived xenograft without obvious adverse reactions.

## Conclusion

Overcoming the resistance of conventional chemotherapeutic drugs and reducing their adverse reactions, is a major challenge in the treatment of cancer. In summary, taking the advantages of cyclic disulfide-bridged peptides, we have developed a GSH/CB dual-controlled precursor of a cyclic disulfide-bridged peptide, cyclic-1a, that formed a supramolecular hydrogel for overcoming DOX-resistance in breast cancer. Cyclic-1a showed high anticancer efficacy against DOX-resistant breast cancer cells *in vitro* and *in vivo*, with no adverse reactions. Therefore, cyclic-1a might be used as a potential chemotherapeutic agent for DOX-resistant breast cancer. This work confirms that the GSH/CB dual-controlled peptide nanostructure provide a useful approach for generating superior functional biomaterials in more precise and controllable modes. In addition, this GSH/CB dual-controlled nanostructure might deliver multiple types of therapeutic drugs and/or diagnostic agents (e.g., small organic molecules, peptides, antibodies, and nucleic acids) through the same carrier to enable combination therapy with a potential to overcome drug resistance, and real-time readout on the treatment efficacy. Our study might provide a useful strategy for the design of supramolecular nanomaterials for drug-resistant cancer diagnostics and therapy. It is anticipated that the precisely dual-controlled nanostructure will emerge as the attractive platform for cancer therapy and many other biomedical applications in cell fate control, drug delivery, regenerative medicine, immune response manipulation, and sensing.

## Data Availability

The original contributions presented in the study are included in the article/Supplementary Material, further inquiries can be directed to the corresponding authors.
