# Supplementary material for: A GSH/CB Dual-Controlled Self-Assembled Nanomedicine for High-Efficacy Doxorubicin-Resistant Breast Cancer Therapy
Source: Front Pharmacol. 2022 Jan 14;12:811724. doi: 10.3389/fphar.2021.811724 (PMC8795745; doi:10.3389/fphar.2021.811724)
Supplement: Supplementary file 1 [file DataSheet1.docx]

**A GSH/CB Dual-Controlled Self-Assembled Nanomedicine for High-Efficacy Doxorubicin-Resistant Breast Cancer Therapy**

Yang Yang^1,^ ^†,^ *, Quanfeng Zhao^3, †^, Zhe Peng^1^, Yunjiang Zhou^4^, Miao-Miao Niu^4^, Lin Chen^1, 2^^,^ *

^1^Department of Pharmacology, Chongqing Health Center for Women and Children, Chongqing 401147, China

^2^Department of Pharmacology, Chongqing Medical University, Chongqing 400016, China

^3^Department of Pharmacy, Southwest Hospital, First Affiliated Hospital to TMMU, Third Military Medical University (Army Medical University), Chongqing 400038, China

^4^Key Laboratory of Drug Quality Control and Pharmacovigilance, Ministry of Education, State Key Laboratory of Natural Medicines, School of Basic Medicine and Clinical Pharmacy, China Pharmaceutical University, Nanjing 210009, China

^†^ These authors contributed equally to this work.

*Correspondence: cqfyyy2020@163.com (Y.Y.); cqfycl@126.com (L.C.); Tel: +86-23-60354448

**Materials and Methods**

**1. Materials**

All the peptides were purchased from GL Biochem (Shanghai) Co., Ltd. Doxorubicin (purity>99%) was purchased from Selleck Chemicals (Houston, USA). Fmoc-protected amino acids and reagents were obtained from GL Biochem (Shanghai, China). Rink-Amide MBHA resin (0.48 mmol g^‒1^) and Wang resin (0.30 mmol g^‒1^) were obtained from Tianjin Nankai Hecheng Science & Technology (Tianjin, China). Cathepsin B, glutathione (GSH) and human serum were purchased from Sigma-Aldrich (St. Louis, MO, USA). Anti-cathepsin B, Anti-caspase-3, anti-Cleaved caspase-3, anti-PARP, and anti-cleaved PARP antibodies were purchased from Cell Signaling Technology (Danvers, USA). Anti-actin and anti-Ki67 antibodies were purchased from Bioworld (Minnesota, USA).

**2. Molecular self-assembly**

Molecular self-assembly was performed as described previously.^[1]^ Briefly, the cyclic-1a was diluted into the phosphate-buffered saline (PBS; pH 7.4) to a final concentration of 1600 µM and incubated at 37 °C. The GSH (8 mM) was then added to the above solution for 12 h at 37 ºC to trigger the first step of self-assembly. Afterward, cathepsin B was added to the solution for 12 h at 37 ºC to trigger the second step of self-assembly (1 U/mL).

**2.1. The first step self-assembly of cyclic-1a to linear-1a**

According to the same method as part 3, 2 mL of the samples were prepared for this experiment. After adding GSH, 100 µL of each sample was collected at each time point. The conversion ratio was then analyzed by LC-MS. The conversion ratio was obtained by integrating the peaks area.

**2.2. The second step self-assembly of linear-1a to linear-1b**

2 mL of the sample prepared by part 3 were adding GSH (8 mM) for 12 h, and then added 1 U/mL of cathepsin B, 100 µL of each sample was collected at each time point and 100 µL methanol was added to terminate the reaction. The method of detection was similar to part 3.1.

**3. Microscopy**

Microscopy was performed as described previously.^[2,3]^ Briefly, hydrogel (5~10 µL) was put onto a carbon support film (20~25 nm) on a copper grid. Excess solvent was carefully removed by filter paper and immediately stained with phosphotungstic acid for 2 min. The grids were allowed to dry for 10 min. The morphology was observed by transmission electron microscope (TEM, Hitachi, H-7650, Tokyo, Japan). Hydrogel (5~10 µL) was dropped on freshly cleaved mica. The characteristic analysis of surface morphology was performed by atomic force microscopy (AFM, Bruker Dimension Icon VT-1000, Santa Barbara, CA, USA) in a tapping mode. Hydrogel (100 µL) was placed in a 24-well culture plates. Gels were dehydrated in a series of ethanol-water solutions, progressing from 30% to 100% ethanol over the course of 24 h. The dehydrated gels were critical point dried using an Electron Microscopy Sciences 850 critical point drier. The morphology was observed by a scanning electron microscopy (SEM, FEI-quanta 400, USA). The conversion ratio was analyzed by LC-MS. The conversion ratio was obtained by integrating the peaks area.

**4. Rheological measurement**

Rheological measurement was performed as described previously.^[2]^ Briefly, rheological tests were conducted on a rheometer (Anton Paar, MCR302, Austria) with 25 mm parallel plate geometry during the experiment. After GSH and CB were added at room temperature, the resulting solution (2 mL) was transferred to the rheometer. The mode of dynamic time sweep was used at strain of 0.1% and frequency of 6.28 rad/s.

**5. Serum stability assay**

Serum stability assay was performed as described previously.^[4]^ Briefly, the supernatant of 25% diluted human serum was collected by centrifugation (15,000 rpm, 10 min). Then, the test peptides were added to the supernatant to obtain a solution with a final peptide concentration of 5 μM. The mixture was incubated at 37 °C. At different time points (0‒360 min), 200 μL of reaction solution was sampled and mixed with 50 µL of 15% trichloroacetic acid and 200 μL acetonitrile. Finally, the supernatant was collected by centrifugation and analyzed by reversed-phase HPLC. Integrated area underneath the peptide peak (monitored at 214 nm) was used for determining the amount of remaining peptide (%).

**6.** **Cellular uptake experiment**

Cellular uptake experiment was performed as described previously.^[3]^ Briefly, MCF-7/DOX cells were seeded onto six-well plates (2 × 10^5^ cells/well). After incubation under an atmosphere of humidified 5% CO_2_ at 37 °C for 24 h, the original medium was removed, followed by adding fresh DMEM medium containing 10 µM of FITC-cyclic-1a. After incubation for 0, 3 or 6 h, the culture medium was discarded and the cells were washed with PBS for 3 times followed by fixing with 4% paraformaldehyde solution for 15 min. The nuclei were counterstained with DAPI and the cells were imaged with inverted fluorescence microscope (Nikon, Japan).

**7.** **Determination of intracellular glutathione**

Determination of intracellular glutathione was performed as described previously.^[5]^ Briefly, the cell lysate was prepared in distilled water containing 0.1% deoxycholic acid plus 0.1% sucrose by four cycles of freeze–thaw and centrifuged at 10,000 × g for 10 min at 4 °C. For intracellular GSH, supernatant was precipitated in 1% perchloric acid and centrifuged at 10,000 × g for 5 min at 4 ℃. 20 μL of protein precipitated sample was mixed with 160 μL of 0.1 M phosphate-5 mM EDTA buffer, pH 8.3 and 20 μL o-phthalaldehyde (1 mg/mL in methanol) in a black 96-well culture plates. After 2.5 h of incubation at room temperature in the dark, fluorescence was measured by using multi-function microplate reader at 460 nm. Results are expressed as GSH nmol/mg protein.

**8. MTT assay**

MTT assay was performed as described previously.^[6]^ Briefly, the cells (5 × 10^3^ cells/well) were seeded in 96-well culture plates (Coring) and incubated overnight. Then cells were exposed to various concentrations of cyclic-1a and incubated at 37 °C for 72 h. After that, MTT stock solution (0.5 mg/mL) was added into each well and cultured for additional 4 h. The MTT treated cells were fixed with 150 μL of DMSO. The absorbance in each individual well was measured by using a microplate spectrophotometer at 570 nM (SpectraMax M2e, Molecular Devices Inc. USA).

**9. Gelation tests of cell lysate**

Gelation tests of cell lysate was performed as described previously.^[1]^ Briefly, MCF-7/DOX and MCF-7 cells in 100 mm tissue culture dishes were incubated with cyclic-1a (1600 µM) at 37 °C for 24 h. After collecting the cells and using centrifuge to remove the culture solution, MCF-7/DOX and MCF-7 cells were broke by ultrasonication and the formation of hydrogel was observed. The cell lysates were placed onto a carbon support film (20~25 nm) on a copper grid and stained with phosphotungstic acid for observation of transmission electron microscope (TEM, Hitachi, H-7650, Tokyo, Japan).

**10.** **Cell apoptosis assay**

Cell apoptosis assay was performed as described previously.^[7]^ Briefly, MCF-7/DOX cells were seeded in 6-well culture plates (5 × 10^5^ cells/well) and incubated for 24 h. Various concentrations (0, 25, 50 and 100 μM) of cyclic-1a were added into the 6-well culture plates. After incubated for 72 h, the cells (1 × 10^5^ cells) were collected and washed twice with ice-cold PBS. Afterwards, the cells were resuspended gently in 500 μL of binding buffer, stained with Annexin V-FITC and PI dye according to the manufacturer’s instructions. Cell apoptosis was analyzed using an Accuri C6 flow cytometer (BD Biosciences, MI, USA).

**11. Western blot analysis**

Western blot assay was performed as described previously.^[8]^ Briefly, cells were washed twice with PBS and then lysed in RIPA lysis buffer with 1mM PMSF for 1 h on ice and centrifuged at 13,000 rpm for 30 min at 4°C. The concentrations of total protein were measured by using BCA protein assay kit (Beyotime, Shanghai, China). Proteins were separated on an 8% SDS-PAGE and transferred onto PVDF membrane. The membranes were blocked in 5% bovine serum albumin for 1 h at room temperature and incubated with primary antibodies at 4 °C for overnight. The membranes were then incubated with HRP-conjugated secondary antibodies for 1 h at 37 °C. The signals were analyzed using the ECL chemiluminescence detection system and Image J software (National Institutes of Health, Bethesda, MD, USA) was used to quantify the intensity of the bands.

**12. *In vivo* anticancer activity**

Female BALB/c nude mice (6 weeks old) were purchased from the Experimental Animal Center of Yangzhou University. The experiments involving animals were approved by the Animal Ethics Committee of China Pharmaceutical University (Ethic approval number: 2020-07-003). *In vivo* anticancer activity assay was performed as described previously.^[9]^ Briefly, MCF-7/DOX cells (200 μL, 1 × 10^7^ cells) were implanted into the right mammary fat pad of nude mice. Once tumors grew to 80-100 mm^3^, mice were randomly assigned into four groups and treated intraperitoneally with vehicle, cyclic-1a (20 mg/kg), cyclic-1a (60 mg/kg) or DOX (5 mg/kg) every 3 days for a total of six times. Tumor volume and body weight were measured every 3 days. Tumor volume was calculated using the formula (c × c × d)/2 (c, the smallest diameter; d, the largest diameter).

**13. Immunohistochemical analysis and histological examination**

Immunohistochemical stains against Ki67 and cleaved caspase 3 were performed using immunohistochemistry kit (KeyGen, Nanjing, China) according to the manufacturer’s instructions. Mouse organs (heart, liver, spleen, lung and kidney) tissues were fixed in 4% paraformaldehyde at 4 °C for 48 h, and then embedded in paraffin wax, sectioned (5 µm) and processed for H&E staining. Inverted fluorescence microscope was used to photograph all sections.

**14. Statistical analysis**

All results were expressed as mean ± SD. Statistical analysis was performed with the t-test for two groups or one-way ANOVA for multiple groups. P<0.05 was considered to be a statistically significant difference.

**
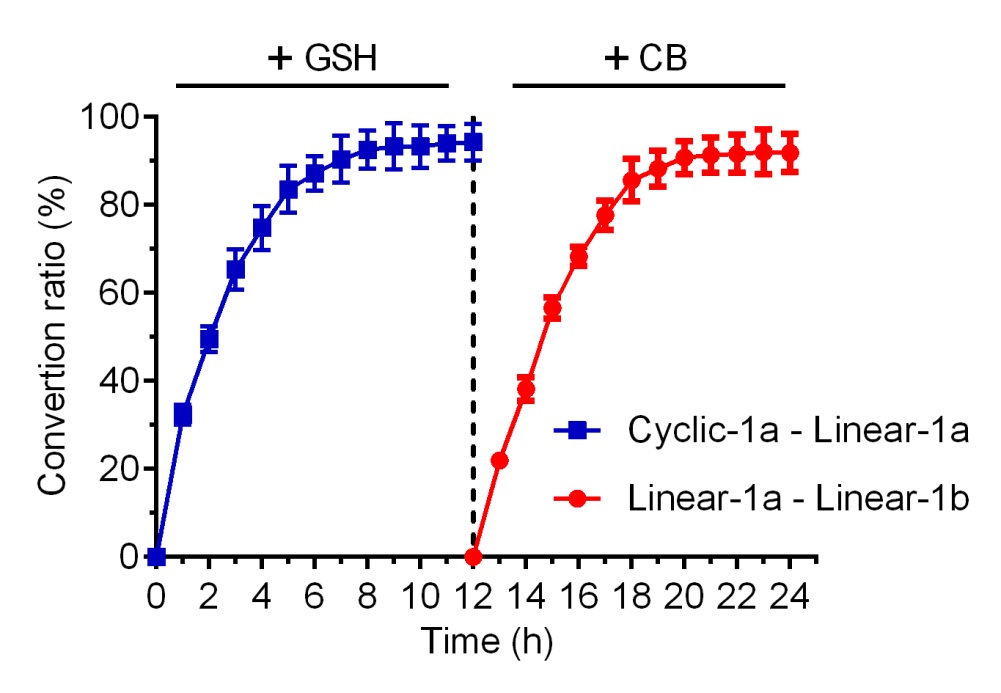
**

**Figure S1.** Conversion ratio of the self-assembly by LC-MS. Data are represented as mean ± SD, n=3.


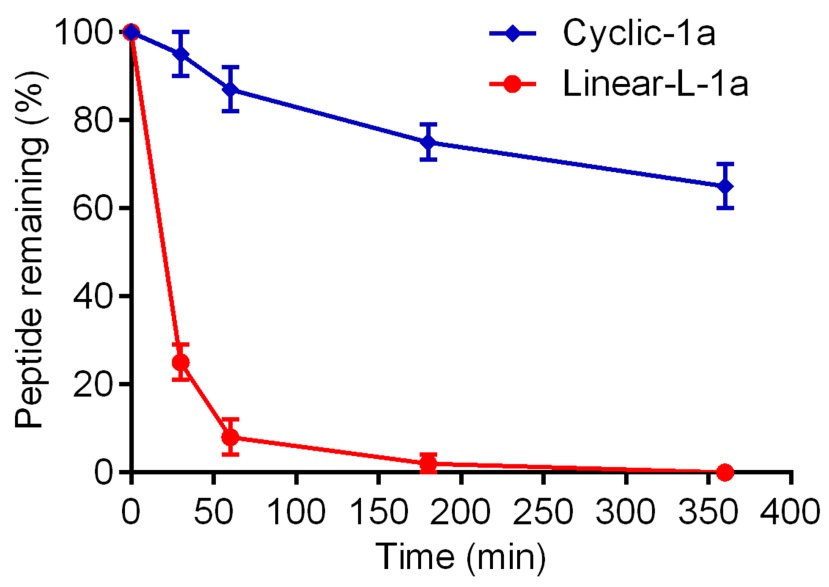


**Figure S2.** Comparison of the proteolytic stability of cyclic-1a and linear-L-1a. Data are represented as mean ± SD, n=3.

**REFERENCES**

1. Zhan, J., Cai, Y. B., He, S. S., Wang, L., and Yang, Z. M. (2018). Tandem Molecular Self-Assembly in Liver Cancer Cells. *Angew. Chem. Int. Ed.* 57, 1813-1816. doi: 10.1002/anie.201710237
2. Yang, Z. M., Xu, K. M., Guo, Z. F., Guo, Z. H., and Xu, B. (2007). Intracellular Enzymatic Formation of Nanofibers Results in Hydrogelation and Regulated Cell Death. *Adv. Mater.* 19, 3152-3156. doi: 10.1002/adma.200701971
3. Yang, D. S., Yang, Y. H., Zhou, Y., Yu, L. L., Wang, R. H., Di, B., and Niu, M. M. (2020). A Redox‐Triggered Bispecific Supramolecular Nanomedicine Based on Peptide Self‐Assembly for High‐Efficacy and Low‐Toxic Cancer Therapy. *Adv. Funct. Mater.* 30, 1904969. doi: 10.1002/adfm.201904969
4. Zhou, Y., Chen, Y., Tan, Y., Hu, R., and Niu, M. M. (2021). An NRP1/MDM2-Targeted D-Peptide Supramolecular Nanomedicine for High-Efficacy and Low-Toxic Liver Cancer Therapy. *Adv. Healthc. Mater.* 10, e2002197. doi: 10.1002/adhm.202002197
5. Akhtar, M. J., Ahamed, M., Alhadlaq, H. A., and Alrokayan, S. A. (2018). MgO nanoparticles cytotoxicity caused primarily by GSH depletion in human lung epithelial cells. *J. Trace Elem. Med Biol.* 50, 283-290. doi: 10.1016/j.jtemb.2018.07.016
6. Zhou, Y., Zhou, Y., Wang, K., Li, T., Yang, M., Wang, R., Chen, Y., Cao, M., and Hu, R. (2020). Flumethasone enhances the efficacy of chemotherapeutic drugs in lung cancer by inhibiting Nrf2 signaling pathway. *Cancer Lett.* 474, 94-105. doi: 10.1016/j.canlet.2020.01.010
7. Zhou, Y., Zhou, Y., Yang, M., Wang, K., Liu, Y., Zhang, M., Yang, Y., Jin, C., Wang, R., and Hu, R. (2019). Digoxin sensitizes gemcitabine-resistant pancreatic cancer cells to gemcitabine via inhibiting Nrf2 signaling pathway. *Redox. Biol.* 22, 101131. doi: 10.1016/j.redox.2019.101131
8. Lin, M. C., Lin, J. J., Hsu, C. L., Juan, H. F., Lou, P. J., and Huang, M. C. (2017). GATA3 interacts with and stabilizes HIF-1α to enhance cancer cell invasiveness. *Oncogene.* 36, 4380. doi: 10.1038/onc.2017.196
9. Zhou, Y., Chen, Y., Huang, X., Tan, Y., Hu, R., Li, C., and Niu, M. M. (2021). A Supramolecular Nanomedicine Based on Bendamustine and MDM2-Targeted D-peptide Inhibitor for Breast Cancer Therapy. *Adv. Healthc. Mater.* 10, e2100980. doi: 10.1002/adhm.202100980
